# Inflammatory Pseudotumor of the Liver Presented in a Patient with Cholelithiasis

**DOI:** 10.7759/cureus.3231

**Published:** 2018-08-29

**Authors:** Eirini V Pantiora, Epameinondas P Sakellaridis, Elissaios A Kontis, Georgios P Fragulidis

**Affiliations:** 1 2nd Department of Surgery, Aretaieio Hospital, National and Kapodistrian University of Athens School of Medicine, Athens, GRC; 2 2nd Department of Surgery, Aretaieio Hospital, The National and Kapodistrian University of Athens School of Medicine, Athens, GRC

**Keywords:** liver, inflammatory pseudotumor, liver surgery, differential diagnosis, liver imaging

## Abstract

An inflammatory pseudotumor of the liver is a rare tumor-like lesion consisting of an inflammatory infiltrate that often can mimic a malignant liver neoplasm. The cause of an inflammatory pseudotumor of the liver is unknown, but it has been reported to be associated with different comorbid conditions most likely inflammatory or infectious in origin. We present an 83-year-old female who presented with a symptomatic gallstones disease and an incidental finding of inflammatory pseudotumor mimicking intrahepatic cholangiocarcinoma in preoperative liver imaging. Differentiating a pseudotumor from hepatic space-occupying neoplasms is crucial since it is one of the most important tumor-mimicking lesions. The imaging findings of this rare tumor can pose diagnostic difficulties because of the amount of fibrosis and cellular infiltration. If malignancy has been excluded, patients can be treated conservatively with steroids and non-steroidal anti-inflammatory drugs. However, complete surgical resection has been the modality of treatment for most of the cases with an indeterminate diagnosis.

## Introduction

An inflammatory pseudotumor (IPT) is a benign and rare process composed of polymorphous inflammatory cell infiltrates and variable amounts of fibrosis. IPT most commonly occurs in the lung, but it can be found in other locations, including the central nervous system, major salivary glands, kidneys, liver, omentum, ovaries, larynx, urinary bladder, breasts, pancreas, spleen, lymph nodes, skin, soft tissues, and orbit of the eye [1-2].

An IPT of the liver is a very rare entity consisting of radiologic features, which are variable and nonspecific. While it may rarely lead to biliary obstruction, portal hypertension, and even cirrhosis, the clinical importance is primarily related to the difficult differential diagnosis from malignant tumors [3]. An IPT of the liver may imitate a malignant tumor on imaging, particularly a metastatic disease or an intrahepatic cholangiocarcinoma (ICC) [3-5]. It is often a solitary tumor with a predilection for the right lobe; however, but it may also manifest as multiple lesions.

IPT of the liver is a challenging diagnosis even with histological criteria since needle biopsy alone may be insufficient. Consequently, some authors suggest surgery as the treatment of choice for patients with severe symptoms or an indeterminate diagnosis [2]. Instead, successful medical treatment among the spontaneous regression of the tumor has been reported [2-5]. As a result, correct recognition of the disease is important. If a diagnosis of IPT of the liver based on a combination of imaging and histological criteria could be achieved, unnecessary surgery may be avoided [3,5]. We discuss herein a female patient who presented with gallstones disease and an incidental finding of a liver mass on preoperative imaging studies, mimicking cholangiocarcinoma. The patient underwent cholecystectomy and liver resection, and the final pathology results of the liver specimen showed an IPT of the liver.

## Case presentation

**Figure 1:**
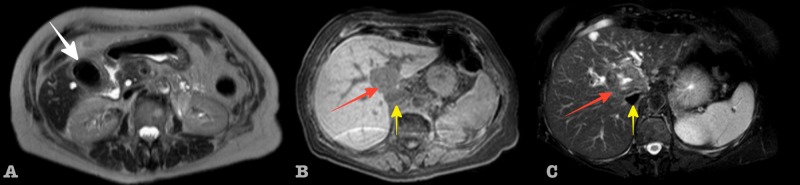
Non-enhanced axial MR image of the upper abdomen: (A) large gallbladder stone (white arrow). T1W SPIR (B) and T2W FS (C) images showing a liver mass (red arrow) adjacent to the IVC (yellow arrow) MR: magnetic resonance: T1W SPIR: T1-weighted spectral presaturation with inversion recovery; T2W FS: T2-weighted fat saturated; IVC: inferior vena cava

An 82-year-old female patient with a symptomatic gallstones disease and a recent weight loss was admitted to our hospital. The patient’s past medical history was free of other diseases and on physical examination, a Murphy sign was present. The abdominal ultrasound mentioned a large gallstone in the gallbladder and a hypoechoic liver mass. Liver blood tests, including tumor markers CEA and CA 19-9 were normal. Magnetic resonance imaging-magnetic resonance cholangiopancreatography (MRI-MRCP) revealed a liver tumor mass (4.5x3.5 cm) located mainly in segments IVa and VIII of the liver with an extent to segment I (Figure 1).

**Figure 2:**
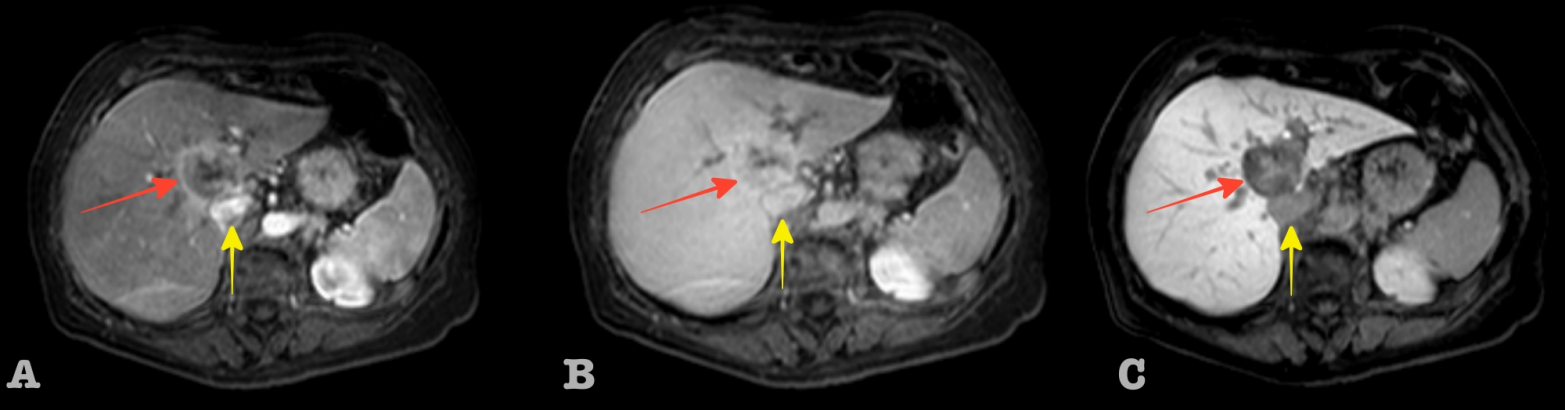
Sequential dynamic contrast enhanced liver MR images. Inflammatory pseudotumor of the liver (red arrow), IVC (yellow arrow): (A) arterial phase 35 sec, (B) portal phase 60 sec., and (C) hepatobiliary phase 30 min. MR: magnetic resonance:  IVC: inferior vena cava

The tumor displaced the adjacent hepatic veins and the inferior vena cava (IVC) without any signs of vessel invasion. There were no signs of liver cirrhosis and no dilated bile ducts or capsular retraction were noted. There was no associated lymphadenopathy. At this point, imaging characteristics were controversial regarding diagnosis. The differential diagnosis tilted in favor of ICC, mainly due to the enhancement characteristics and the absence of liver cirrhosis, as seen in Figure 2.

**Figure 3:**
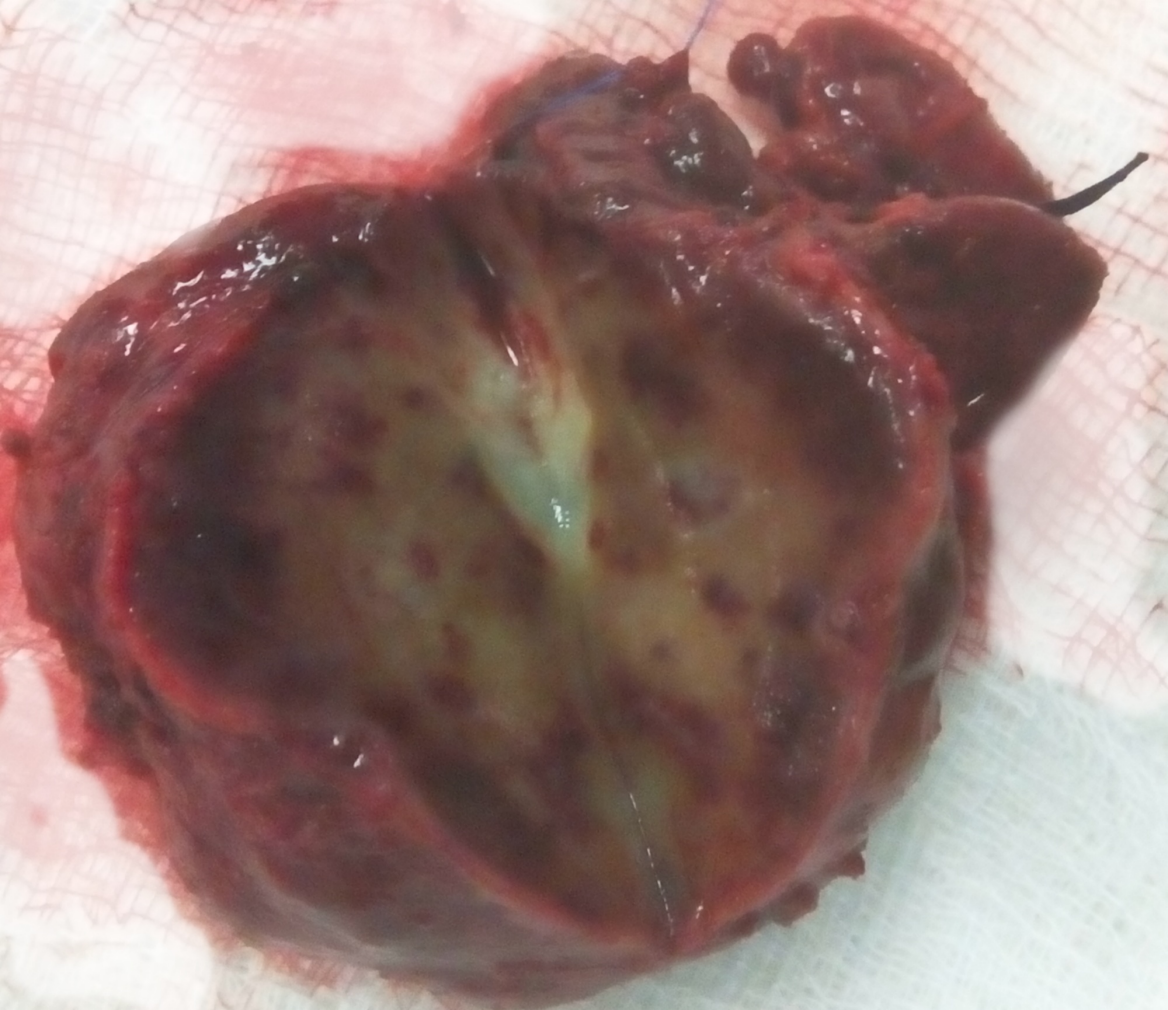
Gross morphology of the liver specimen. In the center, a light yellow region is noted.

The patient was scheduled for exploratory laparotomy with a provisional diagnosis of an ICC. Intraoperatively, a cholecystectomy and lymph node sampling from the hepatoduodenal ligament were performed and both specimens were negative for malignancy on frozen section. Next, the liver was mobilized and the tumor was carefully dissected free of the hepatic veins, the IVC, and the rest of the liver parenchyma. The gross morphology of the liver specimen revealed a solid, grey-yellow liver lesion with a soft consistency. In the center, a light yellow region was noted, as can be seen in Figure 3.

The frozen section was negative for malignancy. Histologically, the tumor was characterized by a heavy inflammatory infiltrate in myxoid collagen stroma, consisting primarily of plasma cells, lymphocytes, and eosinophils. Fibroblast cells without significant fibrosis composed the stroma. There was no evidence of malignancy in the tissue examined. The final pathology report revealed an IPT of the liver.

## Discussion

The term “inflammatory pseudotumor” describes a heterogeneous group of mass-forming lesions that can involve many organs. The lesions are characterized by a prominent inflammatory infiltrate, namely, plasma cells, lymphocytes, and eosinophils as the predominant cellular component [4]. IPT was first described in the lungs by Brunn in 1939 [6]. In 1953, Pack and Backer published the first case occurring in the liver [7]. In 1954, Umiker and Iverson created the term “inflammatory pseudotumor,” as the clinical and imaging findings of the lesion mimic those of malignant tumors [8]. The tumor has been described under several terms and/or variants in the literature: plasma cell granuloma, inflammatory myofibrohistiocytic proliferation, fibroxanthoma, and xanthogranuloma. Several pathologies previously considered to be variants of IPTs have been now identified as different entities because of recent histopathologic, molecular, and cytogenetic advances [9-10]. Currently, some of these tumors represent true neoplasms and, according to an update based on the new World Health Organisation (WHO) classification, may recur or metastasize in as many as 5% of cases [9,11-12]. The neoplastic variants of IPTs include the inflammatory myofibroblastic tumor (IMT) associated with anaplastic lymphoma kinase (ALK-1) translocation, as well as the inflammatory pseudotumor-like follicular dendritic cell tumors of the liver and spleen that are related to a clonal Epstein-Barr virus infection. IMT is a real neoplasm owing to the proliferation of myofibroblastic cells, which occurs mainly in children and young adults and more often involves the lung. IPT is a more inflammatory reactive or regenerative entity and tends to occur more frequently in the liver and affects older patients [10-11].

The etiology and pathogenesis of inflammatory pseudotumors remain unclear for a variety of the rest tumor-like inflammatory lesions that lack the features of the entities listed above [1,4,13]. Mycobacterial spindle-cell inflammatory pseudotumors of lymph nodes and tumor-like lesions associated with immunoglobulin (Ig)G-4 are examples of infectious and autoimmune-induced inflammatory pseudotumors, respectively [4]. A subset of possible immune-related IPTs has been also reported in association with IgG-4-related disorders [10,14].

Liver involvement is rare and is often mistaken as a malignant process particularly metastatic disease or ICC. IPT of the liver has been estimated with an incidence of about 0.7% and accounts for 8% of extrapulmonary IPTs [15]. In the majority of cases, IPTs of the liver are most likely inflammatory or infectious in origin. The presence of gallstones could potentially enhance an inflammatory response and possibly an IPT of the liver, as it is presented in our case. However, in cases of pseudotumors of the liver with inflammatory myofibroblastic features, such as IMTs, a malignant course with local recurrences and distant metastasis has been reported in the literature [9,16-17].

Patients with IPT of the liver present with non-specific clinical symptoms, such as abdominal pain, fever, and weight loss. The lesion may be as large as 25 cm and the radiologic features of IPT of the liver are nonspecific, possibly because of the amount of fibrosis and cellular infiltration [18-19]. Despite recent increases in the diagnostic capability of radiologic studies, differentiating IPT of the liver from other focal hepatic lesions is challenging. Ultrasound and computed tomography (CT) scans are not specific, revealing variable patterns of echogenicity or a liver mass [2]. A CT scan usually reveals lesions with variable contrast enhancement and may display a hypovascular pattern because of fibrosis and delayed enhancement similar to metastatic liver tumors and ICC [2]. MRI may produce low signal intensity on T1-weighted images with a moderate to high signal intensity on a T2 sequence [2]. Peripheral enhancement on the delayed phase is commonly described, attributable to the tumor's fibrous component. The mass is surrounded by the fibrocollagenous stroma rich with capillary vessels, which may explain the peripheral enhancement in the portal and delayed phases scans in the majority of cases due to the extravascular accumulation of the contrast medium [19]. Therefore, these findings of enhanced CT and MRI are relevant to the course of the disease, presence of fibrous tissue, and cellular component [17]. Similarly, the uptake value of 18F-FDG on positron emission tomography (PET)/CT is thought to vary according to the proportion of fibrosis and inflammatory cell infiltrate with high FDG uptake among lymphocytes. The 18F-FDG PET/CT descriptions of hepatic IPTs are limited to case reports and abnormal metabolic activities with a high-standardized uptake value of 7.1 and 7.3 have been reported [18]. A definitive diagnosis of IPT can be made based on needle biopsy findings and, occasionally, in-needle aspiration, as long as the pathologist is aware of this possibility [2]. Nevertheless, although a liver biopsy indisputably has a role in the investigation and management of liver metastases of unknown origin, its role is more contentious and possibly dangerous in cases of a solitary hepatic mass that is likely to be malignant. The main histopathological findings in all cases are the presence of myofibroblastic spindle cells, plasma cells, macrophages, and lymphocytes without cellular atypia or atypical mitotic figures [2]. A biopsy of the tumor is not necessary when planning a surgical intervention for the liver.

Treatment is controversial with some authors suggesting surgery as the definitive treatment for patients with severe symptoms or an indeterminate diagnosis. Other multicenter studies, however, have reported good outcomes after conservative management with antibiotics and/or corticosteroids, but some of these lesions recurred [2,18-19]. However, even though an IPT of the liver may spontaneously regress or regress following antibiotic treatment, a common practice to excise a resectable liver tumor in the absence of a firm diagnosis sounds reasonable. This approach is preferable because it minimizes the risk of a biopsy-related complication, such as dissemination in cases of malignancy; it eliminates the possibility of an IPT recurrence; and, finally, it aims at the pathology diagnosis.

## Conclusions

The absence of the typical features of malignancy on imaging in a liver mass should prompt the consideration of an alternative diagnosis of rare liver lesions, such as an inflammatory pseudotumor of the liver. Differentiating this pseudotumor from hepatic space-occupying lesions is crucial since an inflammatory pseudotumor of the liver may regress spontaneously with conservative management. However, the treatment of choice is still surgical resection for patients with severe symptoms or an indeterminate diagnosis.
